# Acetato­chlorido[2,2′-(ethane-1,2-di­yl)di-1*H*-benzimidazole]­copper(II) monohydrate

**DOI:** 10.1107/S160053681003326X

**Published:** 2010-08-21

**Authors:** Yu-xian Li, Huai-xia Yang, Ya-nan Ding, Xiang-ru Meng

**Affiliations:** a Pharmacy College, Henan University of Traditional Chinese Medicine, Zhengzhou 450008, People’s Republic of China; b Department of Chemistry, Zhengzhou University, Zhengzhou 450052, People’s Republic of China

## Abstract

In the title complex, [Cu(CH_3_COO)Cl(C_16_H_14_N_4_)]·H_2_O, the Cu^II^ ion is five-coordinated by two N atoms from a 2,2′-(ethane-1,2-di­yl)di-1*H*-benzimidazole ligand, two O atoms from a chelating acetate ligand and one terminal monodentate Cl atom in a distorted square-pyramidal geometry. In the crystal, adjacent mol­ecules are linked through O—H⋯Cl, N—H⋯Cl, N—H⋯O and O—H⋯O hydrogen bonds into a three-dimensional network.

## Related literature

1,2-Bis(2,2′-1*H*-benzimidazol)ethane (bbe) has been extensively used in the construction of complexes since it has multiple nitro­gen donors which show strong coordination ability, see: Yang *et al.* (2010 ▶); van Albada *et al.* (2000 ▶). For the potential applications of copper complexes, see: Mirica *et al.* (2004 ▶); Zhang *et al.* (2008 ▶).
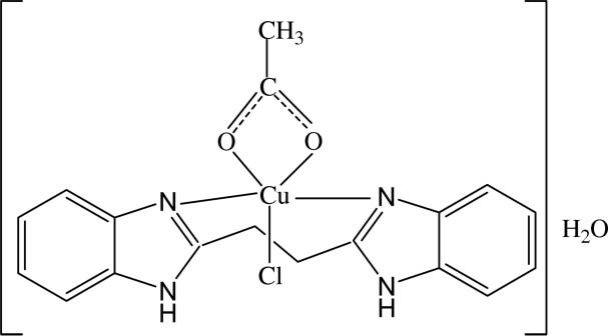


## Experimental

### 

#### Crystal data

[Cu(C_2_H_3_O_2_)Cl(C_16_H_14_N_4_)]·H_2_O
*M* *_r_* = 438.36
Monoclinic, 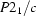

*a* = 14.796 (3) Å
*b* = 8.5844 (17) Å
*c* = 15.162 (3) Å
β = 108.04 (3)°
*V* = 1831.2 (6) Å^3^
*Z* = 4
Mo *K*α radiation
μ = 1.37 mm^−1^
*T* = 293 K
0.22 × 0.20 × 0.19 mm

#### Data collection

Rigaku Saturn diffractometer
Absorption correction: multi-scan (*CrystalClear*; Rigaku/MSC, 2006 ▶) *T*_min_ = 0.753, *T*_max_ = 0.781
12276 measured reflections
3573 independent reflections
3135 reflections with *I* > 2σ(*I*)
*R*_int_ = 0.029

#### Refinement

*R*[*F* ^2^ > 2σ(*F* ^2^)] = 0.040
*wR*(*F* ^2^) = 0.101
*S* = 1.07
3573 reflections
244 parameters
H-atom parameters constrained
Δρ_max_ = 0.37 e Å^−3^
Δρ_min_ = −0.33 e Å^−3^

### 

Data collection: *CrystalClear* (Rigaku/MSC, 2006 ▶); cell refinement: *CrystalClear*; data reduction: *CrystalClear*; program(s) used to solve structure: *SHELXS97* (Sheldrick, 2008 ▶); program(s) used to refine structure: *SHELXL97* (Sheldrick, 2008 ▶); molecular graphics: *SHELXTL* (Sheldrick, 2008 ▶); software used to prepare material for publication: *SHELXTL*.

## Supplementary Material

Crystal structure: contains datablocks global, I. DOI: 10.1107/S160053681003326X/br2145sup1.cif

Structure factors: contains datablocks I. DOI: 10.1107/S160053681003326X/br2145Isup2.hkl

Additional supplementary materials:  crystallographic information; 3D view; checkCIF report

## Figures and Tables

**Table 1:** Hydrogen-bond geometry (Å, °)

*D*—H⋯*A*	*D*—H	H⋯*A*	*D*⋯*A*	*D*—H⋯*A*
O3—H1*W*⋯Cl1	0.82	2.32	3.132 (2)	173
N2—H2*A*⋯Cl1^i^	0.86	2.44	3.203 (2)	149
N4—H4*A*⋯O3^ii^	0.86	1.93	2.786 (3)	172
O3—H2*W*⋯O1^iii^	0.82	2.00	2.822 (3)	176

Symmetry codes: (i) 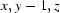; (ii) 
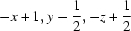
; (iii) 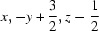.

**Fig. 1.**
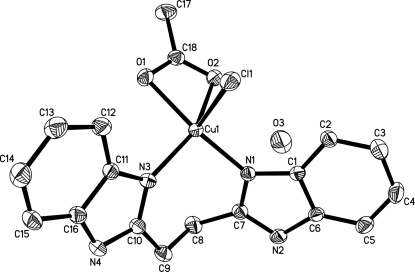
View of the title complex, showing the labeling of the 30% probability ellipsoids. H atoms are omitted for clarity.

## supplementary crystallographic information

### Comment

1,2-Bis(2,2'-1*H*-benzimidazol)ethane (bbe) has been extensively used in the construction of complexes since it has multiple nitrogen donors which show strong coordination ability (Yang *et al.*, 2010; Albada *et al.*, 2000). In addition, copper complexes have received much more attention owing to their potential applications in catalysis, magnetism, electrical conductivity, optical materials and so on. (Mirica *et al.*, 2004; Zhang *et al.*, 2008). In this work, through the reaction of 1,2-bis(2,2'-1*H*-benzimidazol)ethane hydrochloride with copper acetate at room temperature, we obtained the title complex [Cu(bbe) (C H_3_C O O) (Cl)]H_2_O, which is reported here.

In the title complex, each Cu^II^ ion is five coordinated by two oxygen atoms from a chelating acetate ligand, two nitrogen atoms from a bbe ligand and one terminal monodentate Cl atom. Atoms N1, N3, O1, O2 and Cu1 are nearly co-planar (the mean deviation from plane is 0.1042 Å). Cl1 atom is located in the apical position. So the local environment around the central Cu^II^ ion can be best described as a distorted square pyramidal geometry.(Fig.1).  In addition, there are four kinds of hydrogen bonds in the solid state. (*a*) O—H···Cl hydrogen bond between uncoordinated water and Cl atom, (*b*) N—H···Cl hydrogen bond between bbe ligand and Cl atom, (*c*) N—H···O hydrogen bond between bbe ligand and uncoordinated water, (*d*) O—H···O hydrogen bond between uncoordinated water and acetate group. [Cu(bbe) (C H_3_C O O) (Cl)] H_2_O units are linked through these hydrogen bonds resulting in a three-dimensional packing structure in solid state.

### Experimental

The ligand 1,2-bis(2,2'-1*H*-benzimidazol)ethane hydrochloride (0.1 mmol) in methanol (5 ml) was added dropwise to an aqueous solution (2 ml) of copper acetate (0.1 mmol). The resulting solution was allowed to stand at room temperature. After two weeks green crystals with good quality were obtained from the filtrate and dried in air.

### Refinement

H atoms are positioned geometrically and refined as riding atoms, with C-H = 0.93 (aromatic), 0.97 (CH_2_) and 0.96 (CH_3_) Å and O-H = 0.82 Å, and with U_iso_(H) = 1.2 (1.5 for methyl) U_eq_(C,O).

### Crystal data

**Table:**

[Cu(C_2_H_3_O_2_)Cl(C_16_H_14_N_4_)]·H_2_O	*F*(000) = 900
*M**_r_* = 438.36	*D*_x_ = 1.590 Mg m^−^^3^
Monoclinic, *P*2_1_/*c*	Mo *K*α radiation, λ = 0.71073 Å
Hall symbol: -P 2ybc	Cell parameters from 4685 reflections
*a* = 14.796 (3) Å	θ = 2.3–27.9°
*b* = 8.5844 (17) Å	µ = 1.37 mm^−^^1^
*c* = 15.162 (3) Å	*T* = 293 K
β = 108.04 (3)°	Prism, green
*V* = 1831.2 (6) Å^3^	0.22 × 0.20 × 0.19 mm
*Z* = 4	

### Data collection

**Table:**

Rigaku Saturn diffractometer	3573 independent reflections
Radiation source: fine-focus sealed tube	3135 reflections with *I* > 2σ(*I*)
graphite	*R*_int_ = 0.029
Detector resolution: 28.5714 pixels mm^-1^	θ_max_ = 26.0°, θ_min_ = 2.8°
ω scans	*h* = −18→18
Absorption correction: multi-scan (*CrystalClear*; Rigaku/MSC, 2006)	*k* = −10→10
*T*_min_ = 0.753, *T*_max_ = 0.781	*l* = −18→15
12276 measured reflections	

### Refinement

**Table:**

Refinement on *F*^2^	Primary atom site location: structure-invariant direct methods
Least-squares matrix: full	Secondary atom site location: difference Fourier map
*R*[*F*^2^ > 2σ(*F*^2^)] = 0.040	Hydrogen site location: inferred from neighbouring sites
*wR*(*F*^2^) = 0.101	H-atom parameters constrained
*S* = 1.07	*w* = 1/[σ^2^(*F*_o_^2^) + (0.0516*P*)^2^ + 0.5788*P*] where *P* = (*F*_o_^2^ + 2*F*_c_^2^)/3
3573 reflections	(Δ/σ)_max_ < 0.001
244 parameters	Δρ_max_ = 0.37 e Å^−^^3^
0 restraints	Δρ_min_ = −0.33 e Å^−^^3^

### Special details

**Table:**

Geometry. All e.s.d.'s (except the e.s.d. in the dihedral angle between two l.s. planes) are estimated using the full covariance matrix. The cell e.s.d.'s are taken into account individually in the estimation of e.s.d.'s in distances, angles and torsion angles; correlations between e.s.d.'s in cell parameters are only used when they are defined by crystal symmetry. An approximate (isotropic) treatment of cell e.s.d.'s is used for estimating e.s.d.'s involving l.s. planes.
Refinement. Refinement of *F*^2^ against ALL reflections. The weighted *R*-factor *wR* and goodness of fit *S* are based on *F*^2^, conventional *R*-factors *R* are based on *F*, with *F* set to zero for negative *F*^2^. The threshold expression of *F*^2^ > σ(*F*^2^) is used only for calculating *R*-factors(gt) *etc*. and is not relevant to the choice of reflections for refinement. *R*-factors based on *F*^2^ are statistically about twice as large as those based on *F*, and *R*- factors based on ALL data will be even larger.

### Fractional atomic coordinates and isotropic or equivalent isotropic displacement parameters (Å^2^)

**Table:**

	*x*	*y*	*z*	*U*_iso_*/*U*_eq_	
Cu1	0.23644 (2)	0.50951 (3)	0.26280 (2)	0.03006 (13)	
N1	0.19690 (16)	0.3120 (2)	0.19598 (15)	0.0313 (5)	
N2	0.19221 (17)	0.0579 (3)	0.17476 (17)	0.0390 (6)	
H2A	0.2008	−0.0398	0.1871	0.047*	
N3	0.37363 (16)	0.4577 (3)	0.32508 (16)	0.0338 (5)	
N4	0.51000 (17)	0.3474 (3)	0.40310 (16)	0.0398 (6)	
H4A	0.5499	0.2766	0.4306	0.048*	
O1	0.22960 (14)	0.6870 (2)	0.35141 (14)	0.0436 (5)	
O2	0.10300 (14)	0.5737 (2)	0.26343 (13)	0.0402 (5)	
O3	0.34911 (15)	0.6401 (2)	0.00045 (14)	0.0475 (5)	
H1W	0.3208	0.6478	0.0390	0.057*	
H2W	0.3163	0.6933	−0.0425	0.057*	
Cl1	0.23462 (6)	0.70125 (8)	0.13960 (5)	0.0456 (2)	
C1	0.15038 (18)	0.2829 (3)	0.10261 (18)	0.0297 (6)	
C2	0.1067 (2)	0.3812 (3)	0.02902 (19)	0.0371 (7)	
H2B	0.1059	0.4885	0.0370	0.044*	
C3	0.0645 (2)	0.3135 (4)	−0.0563 (2)	0.0472 (8)	
H3A	0.0336	0.3767	−0.1064	0.057*	
C4	0.0669 (2)	0.1537 (4)	−0.0695 (2)	0.0505 (8)	
H4B	0.0395	0.1128	−0.1286	0.061*	
C5	0.1084 (2)	0.0554 (4)	0.0021 (2)	0.0475 (8)	
H5A	0.1095	−0.0517	−0.0067	0.057*	
C6	0.14879 (19)	0.1216 (3)	0.08851 (19)	0.0341 (6)	
C7	0.2186 (2)	0.1743 (3)	0.23568 (19)	0.0347 (6)	
C8	0.2706 (2)	0.1519 (3)	0.3365 (2)	0.0422 (7)	
H8A	0.2554	0.0498	0.3555	0.051*	
H8B	0.2490	0.2292	0.3721	0.051*	
C9	0.3775 (2)	0.1656 (3)	0.3583 (2)	0.0443 (8)	
H9A	0.4072	0.1164	0.4179	0.053*	
H9B	0.3960	0.1065	0.3121	0.053*	
C10	0.4171 (2)	0.3257 (3)	0.36158 (19)	0.0350 (6)	
C11	0.44533 (19)	0.5716 (3)	0.34521 (19)	0.0341 (6)	
C12	0.4432 (2)	0.7294 (3)	0.3244 (2)	0.0425 (7)	
H12A	0.3869	0.7788	0.2916	0.051*	
C13	0.5285 (2)	0.8104 (4)	0.3544 (2)	0.0510 (8)	
H13A	0.5290	0.9162	0.3412	0.061*	
C14	0.6132 (2)	0.7376 (4)	0.4037 (2)	0.0531 (9)	
H14A	0.6688	0.7961	0.4229	0.064*	
C15	0.6167 (2)	0.5823 (4)	0.4248 (2)	0.0477 (8)	
H15A	0.6733	0.5334	0.4574	0.057*	
C16	0.5313 (2)	0.5015 (3)	0.3947 (2)	0.0373 (7)	
C17	0.0792 (2)	0.7964 (4)	0.3494 (3)	0.0570 (9)	
H17A	0.0632	0.7570	0.4020	0.086*	
H17B	0.0221	0.8147	0.2992	0.086*	
H17C	0.1137	0.8923	0.3659	0.086*	
C18	0.1398 (2)	0.6794 (3)	0.32005 (19)	0.0370 (7)	

### Atomic displacement parameters (Å^2^)

**Table:**

	*U*^11^	*U*^22^	*U*^33^	*U*^12^	*U*^13^	*U*^23^
Cu1	0.0287 (2)	0.0265 (2)	0.0330 (2)	0.00279 (13)	0.00674 (14)	−0.00318 (13)
N1	0.0321 (12)	0.0269 (12)	0.0326 (12)	−0.0002 (10)	0.0068 (10)	0.0016 (9)
N2	0.0431 (15)	0.0224 (12)	0.0477 (15)	−0.0004 (11)	0.0084 (12)	0.0008 (11)
N3	0.0293 (13)	0.0349 (13)	0.0367 (13)	0.0051 (10)	0.0094 (10)	−0.0012 (10)
N4	0.0310 (13)	0.0421 (14)	0.0404 (14)	0.0097 (11)	0.0026 (10)	0.0034 (11)
O1	0.0339 (12)	0.0483 (13)	0.0463 (12)	0.0038 (9)	0.0093 (9)	−0.0152 (10)
O2	0.0349 (11)	0.0407 (11)	0.0425 (11)	0.0016 (9)	0.0083 (9)	−0.0051 (10)
O3	0.0454 (13)	0.0499 (13)	0.0435 (12)	0.0003 (10)	0.0083 (10)	0.0003 (10)
Cl1	0.0658 (5)	0.0305 (4)	0.0414 (4)	−0.0017 (3)	0.0180 (4)	0.0043 (3)
C1	0.0261 (14)	0.0266 (13)	0.0374 (14)	−0.0026 (11)	0.0111 (11)	−0.0025 (11)
C2	0.0356 (16)	0.0307 (15)	0.0403 (16)	−0.0019 (12)	0.0050 (12)	0.0024 (12)
C3	0.0442 (19)	0.0492 (19)	0.0420 (17)	−0.0032 (15)	0.0044 (14)	0.0054 (14)
C4	0.051 (2)	0.056 (2)	0.0385 (17)	−0.0067 (16)	0.0050 (14)	−0.0139 (15)
C5	0.0467 (19)	0.0378 (17)	0.055 (2)	−0.0022 (15)	0.0107 (15)	−0.0136 (15)
C6	0.0318 (15)	0.0290 (14)	0.0405 (15)	0.0011 (12)	0.0096 (12)	−0.0041 (12)
C7	0.0343 (16)	0.0301 (15)	0.0381 (15)	−0.0011 (12)	0.0088 (12)	0.0032 (12)
C8	0.0473 (19)	0.0364 (16)	0.0405 (16)	−0.0020 (14)	0.0098 (14)	0.0107 (13)
C9	0.0460 (19)	0.0359 (16)	0.0437 (17)	0.0075 (14)	0.0031 (14)	0.0076 (13)
C10	0.0330 (15)	0.0387 (16)	0.0319 (14)	0.0058 (12)	0.0081 (12)	0.0003 (12)
C11	0.0264 (14)	0.0387 (16)	0.0363 (15)	0.0014 (12)	0.0082 (11)	−0.0053 (12)
C12	0.0344 (16)	0.0404 (17)	0.0521 (18)	0.0016 (13)	0.0125 (14)	−0.0040 (14)
C13	0.0449 (19)	0.0432 (19)	0.068 (2)	−0.0056 (15)	0.0222 (17)	−0.0068 (16)
C14	0.0349 (18)	0.063 (2)	0.064 (2)	−0.0098 (16)	0.0190 (16)	−0.0125 (18)
C15	0.0281 (16)	0.059 (2)	0.0528 (19)	0.0062 (15)	0.0073 (13)	−0.0038 (16)
C16	0.0311 (15)	0.0444 (18)	0.0364 (15)	0.0049 (13)	0.0107 (12)	−0.0034 (13)
C17	0.053 (2)	0.056 (2)	0.067 (2)	0.0192 (17)	0.0255 (18)	−0.0051 (18)
C18	0.0407 (17)	0.0375 (16)	0.0349 (15)	0.0069 (13)	0.0150 (13)	0.0021 (13)

### Geometric parameters (Å, °)

**Table:**

Cu1—N1	1.969 (2)	C4—C5	1.362 (4)
Cu1—N3	2.006 (2)	C4—H4B	0.9300
Cu1—O2	2.053 (2)	C5—C6	1.382 (4)
Cu1—O1	2.0546 (19)	C5—H5A	0.9300
Cu1—C18	2.387 (3)	C7—C8	1.496 (4)
Cu1—Cl1	2.4836 (8)	C8—C9	1.517 (4)
N1—C7	1.320 (3)	C8—H8A	0.9700
N1—C1	1.392 (3)	C8—H8B	0.9700
N2—C7	1.335 (4)	C9—C10	1.489 (4)
N2—C6	1.379 (3)	C9—H9A	0.9700
N2—H2A	0.8600	C9—H9B	0.9700
N3—C10	1.336 (3)	C11—C12	1.389 (4)
N3—C11	1.405 (4)	C11—C16	1.397 (4)
N4—C10	1.336 (4)	C12—C13	1.387 (4)
N4—C16	1.375 (4)	C12—H12A	0.9300
N4—H4A	0.8600	C13—C14	1.393 (5)
O1—C18	1.267 (3)	C13—H13A	0.9300
O2—C18	1.252 (3)	C14—C15	1.368 (5)
O3—H1W	0.8200	C14—H14A	0.9300
O3—H2W	0.8199	C15—C16	1.389 (4)
C1—C2	1.389 (4)	C15—H15A	0.9300
C1—C6	1.400 (4)	C17—C18	1.502 (4)
C2—C3	1.378 (4)	C17—H17A	0.9600
C2—H2B	0.9300	C17—H17B	0.9600
C3—C4	1.389 (4)	C17—H17C	0.9600
C3—H3A	0.9300		
			
N1—Cu1—N3	98.48 (9)	N1—C7—N2	112.2 (2)
N1—Cu1—O2	95.78 (9)	N1—C7—C8	123.8 (2)
N3—Cu1—O2	153.13 (9)	N2—C7—C8	123.9 (2)
N1—Cu1—O1	155.25 (9)	C7—C8—C9	112.6 (3)
N3—Cu1—O1	95.98 (9)	C7—C8—H8A	109.1
O2—Cu1—O1	63.62 (8)	C9—C8—H8A	109.1
N1—Cu1—C18	126.68 (10)	C7—C8—H8B	109.1
N3—Cu1—C18	126.69 (10)	C9—C8—H8B	109.1
O2—Cu1—C18	31.63 (9)	H8A—C8—H8B	107.8
O1—Cu1—C18	32.06 (9)	C10—C9—C8	116.9 (2)
N1—Cu1—Cl1	104.57 (7)	C10—C9—H9A	108.1
N3—Cu1—Cl1	105.94 (7)	C8—C9—H9A	108.1
O2—Cu1—Cl1	92.25 (6)	C10—C9—H9B	108.1
O1—Cu1—Cl1	90.52 (7)	C8—C9—H9B	108.1
C18—Cu1—Cl1	90.03 (7)	H9A—C9—H9B	107.3
C7—N1—C1	106.1 (2)	N4—C10—N3	111.7 (3)
C7—N1—Cu1	123.01 (18)	N4—C10—C9	118.8 (2)
C1—N1—Cu1	130.73 (17)	N3—C10—C9	129.4 (3)
C7—N2—C6	108.1 (2)	C12—C11—C16	119.6 (3)
C7—N2—H2A	126.0	C12—C11—N3	131.8 (3)
C6—N2—H2A	126.0	C16—C11—N3	108.5 (2)
C10—N3—C11	105.4 (2)	C13—C12—C11	117.4 (3)
C10—N3—Cu1	132.1 (2)	C13—C12—H12A	121.3
C11—N3—Cu1	122.28 (18)	C11—C12—H12A	121.3
C10—N4—C16	108.8 (2)	C12—C13—C14	121.8 (3)
C10—N4—H4A	125.6	C12—C13—H13A	119.1
C16—N4—H4A	125.6	C14—C13—H13A	119.1
C18—O1—Cu1	88.55 (16)	C15—C14—C13	121.7 (3)
C18—O2—Cu1	89.06 (17)	C15—C14—H14A	119.2
H1W—O3—H2W	102.3	C13—C14—H14A	119.2
C2—C1—N1	132.0 (2)	C14—C15—C16	116.4 (3)
C2—C1—C6	119.8 (2)	C14—C15—H15A	121.8
N1—C1—C6	108.2 (2)	C16—C15—H15A	121.8
C3—C2—C1	117.5 (3)	N4—C16—C15	131.4 (3)
C3—C2—H2B	121.3	N4—C16—C11	105.5 (3)
C1—C2—H2B	121.3	C15—C16—C11	123.1 (3)
C2—C3—C4	121.8 (3)	C18—C17—H17A	109.5
C2—C3—H3A	119.1	C18—C17—H17B	109.5
C4—C3—H3A	119.1	H17A—C17—H17B	109.5
C5—C4—C3	121.5 (3)	C18—C17—H17C	109.5
C5—C4—H4B	119.3	H17A—C17—H17C	109.5
C3—C4—H4B	119.3	H17B—C17—H17C	109.5
C4—C5—C6	117.2 (3)	O2—C18—O1	118.5 (3)
C4—C5—H5A	121.4	O2—C18—C17	121.1 (3)
C6—C5—H5A	121.4	O1—C18—C17	120.4 (3)
N2—C6—C5	132.4 (3)	O2—C18—Cu1	59.32 (14)
N2—C6—C1	105.5 (2)	O1—C18—Cu1	59.39 (14)
C5—C6—C1	122.2 (3)	C17—C18—Cu1	174.9 (2)

### Hydrogen-bond geometry (Å, °)

**Table:**

*D*—H···*A*	*D*—H	H···*A*	*D*···*A*	*D*—H···*A*
O3—H1W···Cl1	0.82	2.32	3.132 (2)	173.
N2—H2A···Cl1^i^	0.86	2.44	3.203 (2)	149.
N4—H4A···O3^ii^	0.86	1.93	2.786 (3)	172.
O3—H2W···O1^iii^	0.82	2.00	2.822 (3)	176.

Symmetry codes: (i) *x*, *y*−1, *z*; (ii) −*x*+1, *y*−1/2, −*z*+1/2; (iii) *x*, −*y*+3/2, *z*−1/2.
